# Development, Feasibility, and Small-Scale Implementation of a Web-Based Prognostic Tool—Surveillance, Epidemiology, and End Results Cancer Survival Calculator

**DOI:** 10.2196/cancer.7120

**Published:** 2017-07-20

**Authors:** Michelle Henton, Bridget Gaglio, Laurie Cynkin, Eric J Feuer, Borsika A Rabin

**Affiliations:** ^1^ Clinical Effectiveness and Decision Science Patient-Centered Outcomes Research Institute Washington, DC United States; ^2^ Office of Advocacy Relations Office of the Director National Cancer Institute Bethesda, MD United States; ^3^ Statistical Research and Applications Branch, Surveillance Research Program Division of Cancer Control and Population Sciences National Cancer Institute Bethesda, MD United States; ^4^ Department of Family Medicine and Public Health School of Medicine University of California San Diego La Jolla, CA United States

**Keywords:** clinical decision-making, communication, neoplasms, patient care team, Internet

## Abstract

**Background:**

Population datasets and the Internet are playing an ever-growing role in the way cancer information is made available to providers, patients, and their caregivers. The Surveillance, Epidemiology, and End Results Cancer Survival Calculator (SEER*CSC) is a Web-based cancer prognostic tool that uses SEER data, a large population dataset, to provide physicians with highly valid, evidence-based prognostic estimates for increasing shared decision-making and improving patient-provider communication of complex health information.

**Objective:**

The aim of this study was to develop, test, and implement SEER*CSC.

**Methods:**

An iterative approach was used to develop the SEER*CSC. Based on input from cancer patient advocacy groups and physicians, an initial version of the tool was developed. Next, providers from 4 health care delivery systems were recruited to do formal usability testing of SEER*CSC. A revised version of SEER*CSC was then implemented in two health care delivery sites using a real-world clinical implementation approach, and usage data were collected. Post-implementation follow-up interviews were conducted with site champions. Finally, patients from two cancer advocacy groups participated in usability testing.

**Results:**

Overall feedback of SEER*CSC from both providers and patients was positive, with providers noting that the tool was professional and reliable, and patients finding it to be informational and helpful to use when discussing their diagnosis with their provider. However, use during the small-scale implementation was low. Reasons for low usage included time to enter data, not having treatment options in the tool, and the tool not being incorporated into the electronic health record (EHR). Patients found the language in its current version to be too complex.

**Conclusions:**

The implementation and usability results showed that participants were enthusiastic about the use and features of SEER*CSC, but sustained implementation in a real-world clinical setting faced significant challenges. As a result of these findings, SEER*CSC is being redesigned with more accessible language for a public facing release. Meta-tools, which put different tools in context of each other, are needed to assist in understanding the strengths and limitations of various tools and their place in the clinical decision-making pathway. The continued development and eventual release of prognostic tools should include feedback from multidisciplinary health care teams, various stakeholder groups, patients, and caregivers.

## Introduction

It comes as no surprise that the Internet has changed the way patients diagnosed with cancer and their caregivers seek information about their diagnosis. The influx of big data and the use of electronic health records (EHR) in the health care system [1] have been instrumental in the evolution of the relationship between large datasets with both patients and providers. Even though the Health Information Technology for Economic and Clinical Health Act (HITECH), which was passed by Congress in 2009 [2,3], is increasing the adoption and use of EHRs, the health care industry as a whole has not been as quick to adopt changes into their systems [4], such as integrating decision support tools or predictive tools (known as nomograms) into physician workflow [5].

The lack of uptake of tools into EHR systems, matched with the increase in tool development as it relates to cancer prognosis, has led to a number of cancer prognostic tools being housed outside of the health care setting. These cancer prognostic tools often use clinical or population datasets (or sometimes a combination of both) to tell a story about millions of patients and their health. On their own, population datasets are overwhelming and not easily understood by the general public. However, when this information is broken down and formatted into tools, large population datasets can give an unbiased estimate about one patient based on the data of millions of others with similar traits. These tools are being developed to allow oncologists to project an individual’s likelihood of cancer recurrence, likely benefit from chemotherapy, probability of mortality at different ages to both improve the accuracy of the oncologist’s knowledge about cancer prognosis and provide a basis for informed decision-making [6], and also allow patients to understand complex health information [7]. This helps open the door for patients to take charge of their own health and gives providers an opportunity to have a conversation with patients and their caregivers about complex health information in a format that is accessible and understandable.

In order to create a Web-based prognostic tool that could draw on the most extensive cancer statistics databases, the National Cancer Institute’s (NCI) Statistics Research and Applications Branch in 2008 developed the Surveillance, Epidemiology and End Results Cancer Survival Calculator (SEER*CSC, formerly known as the Cancer Survival Query System [CSQS]), a prototype of a Web-based prognostic tool. Unlike other tools such as Adjuvant! Online and Memorial Sloan Kettering’s nomograms, which use clinical data [6], SEER*CSC was designed to access SEER and Medicare claims data. SEER*CSC provides physicians with highly valid evidence-based prognostic estimates about cancer to improve the quality of information that physicians have for shared decision-making and risk communication with their patients. The strength of SEER data is that it is population-based, thus providing estimates of survival that may be quite different than patients in clinical trials or seen at major cancer centers [8,9]. The sheer size of the database (from 18 widely different geographic areas representing about 30% of the United States population) ensures that even patients with somewhat uncommon sets of tumor, demographic, and comorbidity profiles can get reasonable estimates of their prognosis. With this, SEER*CSC is a means of making survival estimates from a population-based database more timely, relevant, actionable, understandable, and context-accurate for cancer patients.

The purposes of this study were to: (1) describe the iterative, multistep development and testing of SEER*CSC, (2) discuss lessons learned from a small-scale implementation study, and (3) provide directions for future refinement and release of SEER*CSC.

## Methods

This study consisted of four phases: (1) formative period, (2) provider usability testing, (3) small-scale implementation, and (4) patient usability testing. The institutional review boards at all participating sites approved this study.

### Formative Period

Formative research was conducted in two steps in 2005 and 2008 to develop the prototype of SEER*CSC through the NCI Office of Market Research and Evaluation and a private contractor, User-Centered Design. During this stage of the project, patient advocates were queried about SEER*CSC through usability testing, survey methods, and a focus group. In addition, 7 physicians (1 surgical oncologist, 1 breast surgeon, 2 medical oncologists, 1 urologist, 1 surgeon, and 1 physician of unknown specialty) were interviewed via telephone in 2008 with a structured interview guide, asking about respondents’ background and experience with patients, their experience with similar prognostic tools, and their thoughts and reactions to SEER*CSC approach and intent.

### Provider Usability Testing

Using the knowledge gathered from the formative phase, usability and feasibility data were collected from four health care delivery systems (Kaiser Permanente Colorado, University of Colorado Hospital, Denver Health Medical Center, and Veterans Administration Eastern Colorado Health Care System) on the general applicability, content and design, and implementation potential of SEER*CSC through one-on-one testing sessions with physicians and other members of cancer care teams.

The one-on-one sessions included: (1) semistructured discussion about general and prognosis-specific communication issues with cancer patients (ie, pre-test interview), (2) hands on formal usability testing session using think aloud approach and hypothetical case examples, and (3) semistructured discussion about the applicability and implementation potential of SEER*CSC (ie, post-test interview). The one-on-one sessions were designed to last approximately 90 minutes and were conducted by 1 of 3 members of the research team who were extensively trained in qualitative interviewing, usability testing, and the use and underlying principles of SEER*CSC. We asked 2 medical oncologists from the Dana Farber Cancer Center specializing in prostate and colorectal cancer treatment to review SEER*CSC and develop hypothetical case studies for the usability testing sessions. The one-on-one sessions were audio recorded. The usability portion of the session was recorded using screen capturing software (Camtasia for Mac OS 2010). Interviewers prepared detailed field notes from each session.

### Small-Scale Implementation

Based on the input from the provider usability testing, SEER*CSC was revised. This version was included in a small-scale implementation phase, which consisted of two parts. First, interviews were conducted with 5 physicians from 3 health care delivery systems (Kaiser Permanente Colorado, University of Colorado Hospital, and Denver Health Medical Center). Physician interviewees represented possible site champions for the small-scale implementation study and were knowledgeable on both clinical and information technology barriers and facilitators. All but 1 physician interviewee participated in the previous phase of provider usability testing of SEER*CSC and were familiar with the website. Second, a small-scale implementation of SEER*CSC into 3 specialty care departments (urology, oncology, and surgery) across 4 sites (Kaiser Permanente Colorado, Penrose Cancer Center in Colorado Springs, Colorado, and 2 urology private practices affiliated with Penrose Cancer Center) was conducted. A total of 9 champions were identified and were responsible for the following: (1) meet with study staff to discuss an implementation plan and schedule a time for a roll-out meeting with department staff, where study staff explained the study and demonstrated the tool, (2) distribute a follow-up email created by study staff to their department explaining the study, and (3) participate in a follow-up key informant interview once data collection was complete. Champions were also encouraged to contact study staff when they participated in any follow-up activity, such as discussing SEER*CSC with colleagues, providing a department demonstration, or sending an email/voicemail to colleagues reminding them of SEER*CSC. These activities were documented by study staff to compare with automated usage data.

### Patient Usability Testing

Upon completion of the small-scale implementation study, the possibility of making SEER*CSC patient-facing was considered. To further explore this option, Web-based one-on-one usability testing of SEER*CSC was conducted with patients who were diagnosed with prostate or colorectal cancer. The purpose was to understand health information–seeking practices and preferences around communication of cancer prognostic information to further refine SEER*CSC. Prostate and colorectal cancers were included because they are common cancers often diagnosed at older ages when individuals have significant coexisting conditions. Eligible participants were identified from two advocacy groups: Prostate Cancer, International and Fight Colorectal Cancer. Champions were identified from each advocacy group to inform potential participants about the study and invite them to take part in it through their respective websites. Eligible participants were required to have had a prostate or colorectal cancer diagnosis within the last 5 years (as indicated by self-report from the time of contact). Individuals that responded to the champions’ invitation and were contacted by study staff to set up a time to participate in usability testing.

The one-on-one sessions took place via Cisco WebEx and took approximately 75 minutes. Each session consisted of: (1) informed consent, (2) short survey consisting of demographic questions and questions on prognostic information seeking, (3) formal usability testing, and (4) questions soliciting feedback and recommendations for making SEER*CSC more patient focused. During the formal usability testing portion, participants were asked to enter information into SEER*CSC using case examples developed by the research team. The case examples were matched to patient’s cancer history (eg, participants with prostate cancer history would use a prostate cancer case). Usability sessions were recorded using Cisco WebEx with the permission of the participants.

### Data Analysis

Interviews conducted during the last 3 phases of the study (provider usability testing, small-scale implementation, and patient usability testing) were transcribed verbatim and reviewed against the audio files by a research assistant. Post-interview field notes were saved along with interview transcripts. The narrative data were entered into ATLAS.ti release 6.2 (ATLAS.ti, 2012) for analyses. Data analysis occurred throughout the data collection process. Three interviews were initially coded by 4 members of the research team who created an initial list of codes based on key points in the interview text. The 4 coders then met to discuss codes and create a formal codebook. This process was repeated with 4 additional interviews until the final codebook and thematic framework was created. The remaining interviews were coded with a subset of interviews selected for secondary coding. Comparisons between primary and secondary coders were conducted to assess inter-rater reliability. The findings were deemed to be acceptable using a qualitative comparison of coding patterns across coders and resulted in a 75% agreement. To augment information from the provider and patient usability interview transcripts and field notes, a subset of video files from the Camtasia (for Mac OS 2010) screen recordings were analyzed. A structured abstraction form was used to assess the length of time for which the tool was used per each case study as well as which pages were visited and which features of the tool were used. During the small-scale implementation trial, data were collected electronically on tool usage. Field notes were used to capture champions’ efforts to promote the use of the tool and to help interpret usage data.

## Results

### Overview

The development of SEER*CSC followed an iterative, multistep approach, taking information from each phase into account for the refinement of the tool for the next phase. The results are presented by each phase of the project.

### Formative Period

Formative data collection efforts were conducted in the early development stage of SEER*CSC and suggested potential user perspectives on utility, as well as improvements and safeguards for this prognostic tool to minimize its possible negative consequences. Information gathered by NCI through usability testing, surveys, and a 10 person focus group with patient advocates in 2005 and then again from 9 telephone interviews in 2008 identified similar themes. Patient advocates stated access to survival data is needed, but it must be presented less technically and in such a way as to keep hope alive.

Concern about misuse of prognostic information was noted by advocates. Examples included clinicians who may deny treatment to patients with a low survival rating and insurance companies using the information to ration or deny coverage for treatment. Some advocates further expressed how patients themselves might misuse or misinterpret prognostic information. However, there was consensus among advocates that prognosis information and crude survival data should be available to the patient community and that it would be of use to them. They stated that patients should be able to access any data available to their physicians, and most would use a print-out of SEER*CSC’s results as the basis for dialogue with their clinicians.

Subsequent feedback from physicians and cancer patient advocates on the wireframe of SEER*CSC included: (1) many prognostic tools do not adequately account for comorbidities or account for how treatment affects prognosis, (2) SEER data are less biased than the data relied on by available prognostic tools, and (3) users are not allowed to enter clinically detailed specifications about cancer size and progression. Based on this feedback the initial version of the SEER*CSC calculator was developed.

### Provider Usability Testing

A total of 57 provider interviews were conducted across four health care delivery systems. This included 36 physicians and 21 other types of providers (eg, nurse, pharmacist, and social worker). There was variability in terms of time in current position, with the majority being 1 to 5 years, followed by more than 10 years. Most providers saw cancer patients at least once per day. Demographics of provider interviewees are provided in Table 1.

In terms of usability, SEER*CSC was generally regarded as professional, intuitive, easy-to-use and navigate, and visually appealing. However, there was confusion about how to navigate to previously viewed pages, and that the user agreement and home page needed to be less information dense. Comments were very favorable for the prognostic information sections of the tool. Provider interviewees overwhelmingly felt it was important to include treatment information and the relationship with survival, as this information is key to having the prognosis conversation with their patients (see Table 2).

**Figure 1:**
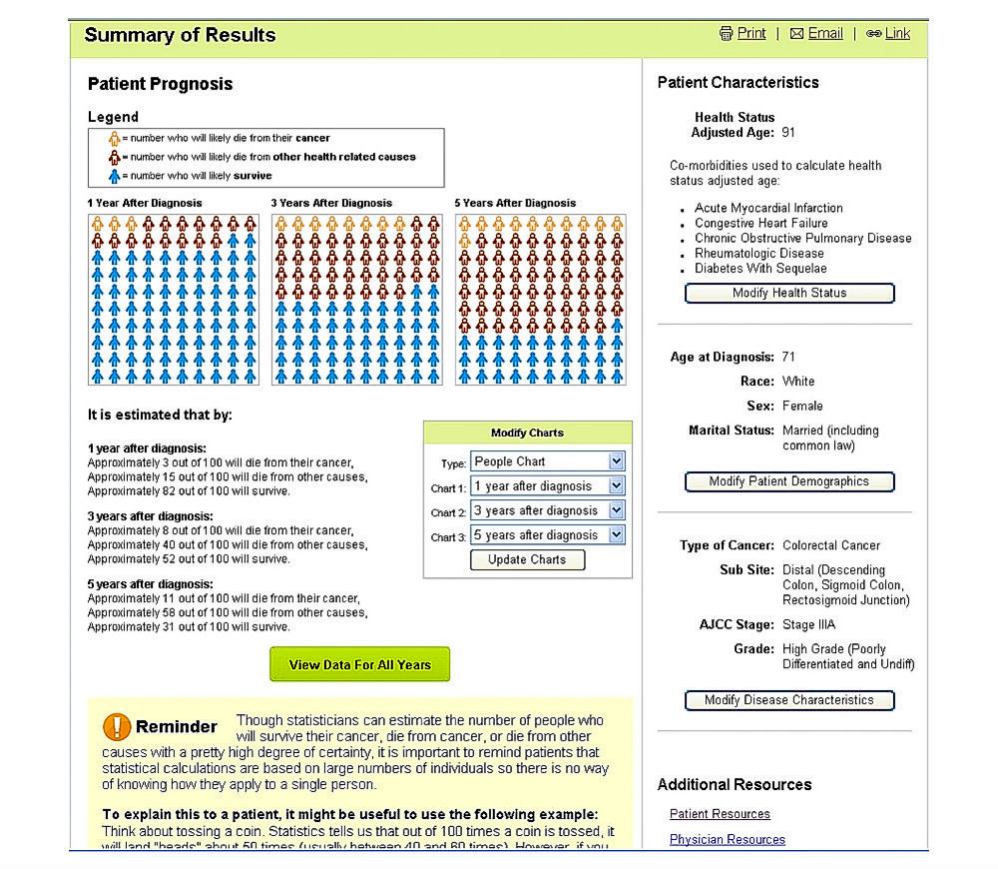
Screenshot of Surveillance, Epidemiology and End Results Cancer Survival Calculator’s (SEER*CSC) Summary of Results page before physician usability testing.

**Figure 2:**
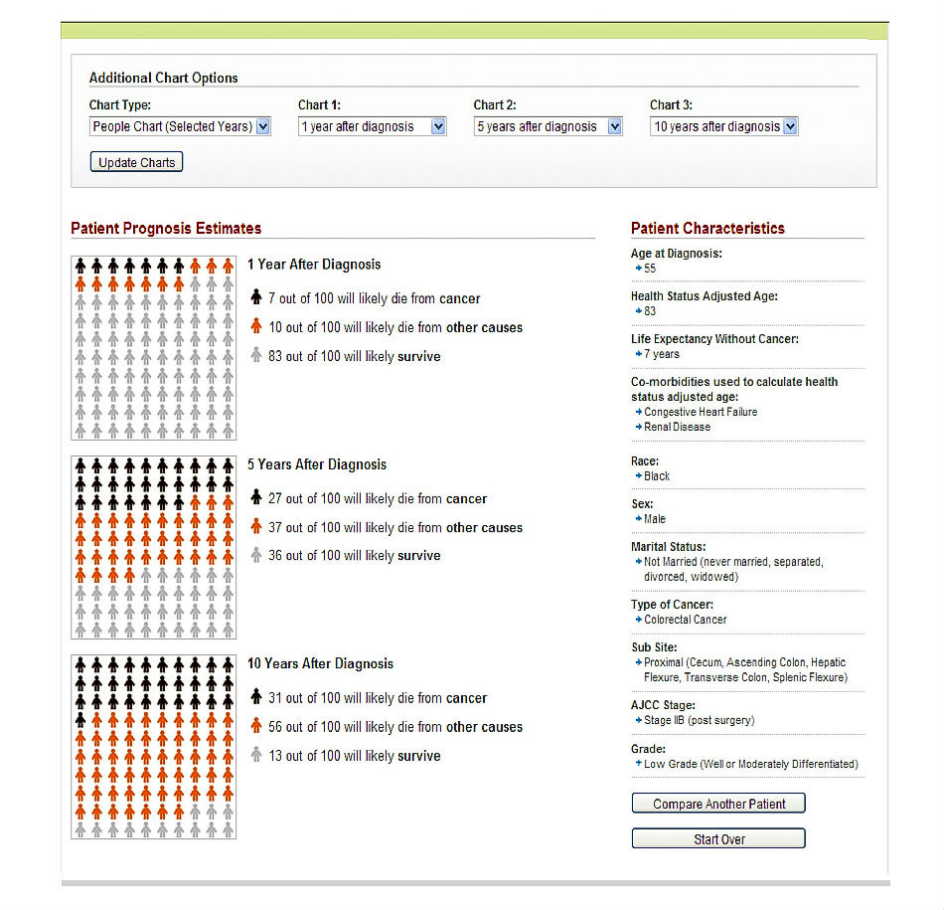
Screenshot of Surveillance, Epidemiology and End Results Cancer Survival Calculator’s (SEER*CSC) Summary of Results page after physician usability testing.

Based on this feedback, changes were made to SEER*CSC (see Figures 1 and 2). The most important changes involved revising the layout of the results page. This included changing the color of the charts to be more distinguishable, adjusting the years after diagnosis to default to 1, 5, and 10 years instead of 1, 3, and 5, and adding a Compare Another Patient feature that allows the user to compare 2 diagnoses using different criteria (eg, age, gender, and comorbid conditions). Additional changes included adding more information in the form of pop-up windows when hovering over a “?” throughout the tool and making the language on the website more concise.

### Small-Scale Implementation

After the physician usability testing, revisions were made to SEER*CSC in preparation for the small-scale implementation. A total of 157 providers (including physicians and nurses) from 7 practices at 4 sites were assigned logins to participate in the implementation of SEER*CSC. Overall, the tool was not widely adopted during the study. Data were tracked from mid-February to mid-May, 2013. During the 3 months of data tracking, usage was low and non-sustained. Table 3 shows that providers had a total of 23 sessions with 45 case scenarios entered, most of which were comparing 1 individual case with multiple modifications (eg, altering demographics, and comorbidities). Attempts to remind champions about contacting providers in their department to use SEER*CSC were unsuccessful. This included up to 2 email reminders that provided language for champions to send to their departments with information about SEER*CSC, the link to the SEER*CSC portal, and a reminder how to log into the portal. Overall implementation of SEER*CSC was not successful.

Exit interviews with the champions revealed that there are no incentives or infrastructure in place for providers to use Web-based prognostic tools. A majority stated they did not use tools when discussing prognosis with their patients because of time and preference/habit. Additional barriers to the implementation of SEER*CSC included not having all the information providers wanted in the tool (eg, treatment), time to enter the data, and not having the tool as part of the electronic health record or readily available on the desktop.

**Table 1:** Demographics of interviewees who participated in the provider usability testing sessions for Surveillance, Epidemiology and End Results Cancer Survival Calculator (SEER*CSC).

Characteristics (N=57)		n (%)
**Gender**		
	Male	21 (37)
	Female	36 (63)
		
**Age group, years**		
	Under 34	10 (21)
	35-44	20 (35)
	45-54	12 (21)
	55-64	14 (25)
	65 and older	1 (2)
		
**Type of provider**		
	Clinical pharmacist	1 (2)
	Nurse practitioner	6 (11)
	Patient navigator/social worker	3 (5)
	Physician	36 (63)
	Physician assistant	2 (4)
	Nurse	7 (12)
	Nurse care coordinator	2 (4)
		
**Specialty**		
	Family medicine	6 (11)
	Internal medicine	8 (14)
	Oncology	17 (30)
	Urology	7 (12)
	Surgery	10 (18)
	Radiology	3 (5)
	Radiation Oncology	2 (4)
	Gastroenterology	2 (4)
	Pharmacy	1 (2)
	Health education	1 (2)
		
**Time in current position**		
	Less than one year	10 (18)
	1-5 years	19 (33)
	6-10 years	10 (18)
	More than 10 years	18 (32)
		
**Time in health care/medicine**		
	0-5 years	7 (12)
	6-10 years	11 (19)
	11-15 years	12 (21)
	More than 15 years	27 (47)
**Frequency of seeing cancer patients**		
	At least once per day	38 (67)
	At least once per week	14 (25)
	Less than once per week	5 (9)

**Table 2:** Summary of combined physician and patient usability testing feedback of Surveillance, Epidemiology and End Results Cancer Survival Calculator (SEER*CSC).

Section	Issue identified	Recommendation
Starting pages	Not all users (especially non-cancer specialist providers) were familiar with SEER (Surveillance, Epidemiology, and End Results).	SEER needs to be better explained (in lay terms) on the home page so users who are not familiar with SEER can also understand the term and reliability of the source. In addition, SEER should be explained on the output pages for those that skip the home page and move right to the calculators.
Prostate disease characteristics	Concerns were raised about the appropriateness of selected categories for Gleason score. Many argued that more recent clinical evidence suggests different categorization of the patients based on their Gleason score. Most suggested three categories with varying cut-off values (eg, 6 and less; 7-8, 9-10).	Categorization of cases based on Gleason score should be reconsidered or existing categorization should be justified.
Prostate disease characteristics	Non-cancer specialists were not always familiar with Gleason score and would have appreciated guidance and definition of the exact clinical meaning and origin of this value. Also, the categories of pre-treatment, pure clinical, and pathologic stage were not intuitive for all interviewees.	Provide definition in the form of pop-up window. It would be important to provide clear explanation on pre-treatment, pure clinical, and pathologic stages since these categories were not always intuitive for interviewees.
Prostate disease characteristics	A few interviewees mentioned that prostate-specific antigen (PSA) values should be added to the algorithm, although one specialist thought that PSA has less impact on prognosis than Gleason score. Several patient users asked why PSA was not included.	While inclusion of PSA values into the algorithm might not be feasible, explanation on why and how the lack of PSA might impact outcomes might increase the trust of providers in the results provided by the tool.
Comorbidity calculator	Many providers expressed general agreement with the accuracy of the health status adjusted age, although several expressed concerns and/or confusion about how adjusted age is calculated and whether interactions or simple additive models are used.	Providing link to the calculations or method used for age adjustment based on comorbidities should be provided.
Comorbidity calculator	Many users wanted to know how the list of comorbidities included in the calculator was selected.	The reason for the choices of conditions in comorbidity calculator should be made more transparently available for users.
Comorbidity calculator	Many providers and patients did not understand why comorbidity data are not available for those under 66, suggesting that an explanation is needed. While some providers knew that the comorbidity calculator is only available for those 66 and over, most did not know the reason for this, and some incorrectly speculated as to the reasons.	It should be more prominently displayed why comorbidity calculator is not available for those under 66.
Summary of results	Print, email, and link functions were regarded as useful services by many interviewees. When testing these functionalities some issues were noted by our research team.	Print, email, link functions need to be thoroughly tested for proper functioning.
Summary of results	A number of users did not note the Modify chart option and needed to be prompted to use this functionality. Furthermore, patient interviewees wanted to see survival data projections beyond 10 years, going up to at least 20 years.	Arrangement of the Summary of Results page should be considered to better differentiate the Update charts and extend survival data calculations up to 20 years.
Additional resources	One consideration might be to continually update and refine the patient and physician resources, particularly as new information becomes available. Providers and patients truly saw the value in having these resources and would appreciate them most if they knew it was the latest and greatest information.	Addition of currently available Web-based prognostic tools and guidance on when to use those (from our systematic review) could be one added resource.

**Table 3:** Data tracking of Surveillance, Epidemiology and End Results Cancer Survival Calculator (SEER*CSC) usability during small-scale implementation in clinical care settings.

Data tracking in clinical care settings		Data pull 1: February 19-April 18, 2013	Data pull 2: April 18-May 17, 2013
Number of case scenarios		30	15
**Type of cancer**			
	Prostate	22	8
	Colorectal	8	7
Total number of individual providers		8^a^	4
**Uses by site**			
	KP Urology	2	2
	KP Oncology	1	0
	KP Surgery	1	0
	Penrose-GI	2	2
	Penrose-Radiation Oncology	1	0
	Private Urology Practice 1	1	0
	Private Urology Practice 2	0	0
Total number of sessions		15^b^	8
**Sessions by site**			
	KP Urology	6	5
	KP Oncology	1	0
	KP Surgery	1	0
	Penrose-GI	4	3
	Penrose-Radiation Oncology	2	0
	Private Urology Practice 1	1	0
	Private Urology Practice 2	0	0

^a^8 individual users signed on to the site; only 7 entered case information.

^b^15 sessions among 8 individual users; only 7 users entered case information.

### Patient Usability Testing

In addition to the small-scale implementation of the SEER*CSC, usability testing and interviews were conducted with patients. A total of 14 individuals completed one-on-one sessions; 7 diagnosed with prostate cancer and 7 diagnosed with colorectal cancer. Patient participants had either completed their course of treatment or were in active surveillance or watchful waiting. Table 4 provides a summary of the patient characteristics. Overall, the reactions to SEER*CSC were positive. Patients felt the Internet was a valuable tool to inform them about their diagnosis and was necessary to help them prepare for conversations with their health care team as they moved through the disease care process.

Patients felt SEER*CSC was easy to navigate, easy to enter the data given, and provided information that would be useful to someone who was recently diagnosed with cancer. Many commented on liking the ability to choose the graphical representation of the results that best meet their needs and ability to understand. They also mentioned liking the additional resources provided. There was some concern as to whether a patient would have the information necessary to complete the disease characteristics section of the tool. The majority commented on the language and terminology used throughout the tool and that it was a limitation to using SEER*CSC. Another major weakness identified was the lack of treatment options in the calculations.

**Table 4:** Demographics of interviewees who participated in the patient usability testing sessions for Surveillance, Epidemiology and End Results Cancer Survival Calculator (SEER*CSC).

Characteristics		Prostate cancer diagnosis	Colorectal cancer diagnosis
**Gender**			
	Male	7	3
	Female		4
**Age, years**			
	35-44		1
	45-54	3	
	55-64	3	4
	65+	1	2
**Race/Ethnicity**			
	Non-white		1
	White	7	7^a^
**Stage at Cancer Diagnosis**			
	Stage I	3	1
	Stage II	1	1
	Stage III	2	2
	Stage IV		3
	Unknown	1	
**Time Since Diagnosis**			
	1 year	1	3
	2 years	1	2
	3 years	2	
	4 years	3	
	5 years		2

^a^Participant identified with two.

## Discussion

### Principal Findings

SEER*CSC is an interactive, Web-based prognostic tool using SEER and linked Medicare data that was developed, tested, and implemented over 4 phases. Overall, providers responded positively to the tool, with some recommended changes, which led to testing it in real-world, clinic settings. Providers expressed their support in patients having access to SEER*CSC. With supplemental funding, patients were given the opportunity to test the tool to gauge whether the information was understandable and whether it was something they would use.

Despite the positive feedback and enthusiasm about the tool, use during the small-scale implementation was low. Lack of utilization of tools is not new in health care settings. Studies have shown that while a number of tools, such as decision aids (DA) and other prognostic, Web-based tools, have increased in development, very few are thoroughly evaluated and/or implemented into routine practice [6,10-13]. Although current studies have shown that these types of tools help patients reduce decisional conflict, increase understanding of diagnosis, and increase patient-provider communication [14,15], there are still many factors that hinder dissemination and implementation (D&I) into real-world, clinical practice.

Based on our study, we postulate the following reasons for low uptake of SEER*CSC. First, the time required to enter necessary data. Clinicians noted that having a tool like SEER*CSC integrated into the EHR system, instead of freestanding, would decrease data entry burden. However, if it remains freestanding, non-physicians, such as nurses and navigators who initially spend time with the patient, could have an opportunity to fill in the data prior to the patient meeting with the physician, thus decreasing time that would otherwise be taken away from patient-physician interaction. Second, SEER*CSC lacks treatment options. Currently, it only provides prognostic information, which is just one part of the conversation physicians and providers have when it comes to cancer diagnosis and treatment. Physicians want to share treatment alternatives with patients. Patients not only want to know what their treatment options are, but how it will affect their prognosis, and then discuss those treatment options with their provider. However, currently no single tool provides everything. Third, providers know the prognostic information needed to communicate with their patient, hence they do not rely on tools. Even though development of DAs are increasing, it is not yet commonplace for physicians to use them in their practice, know they have been developed and tested, or have easy access to them in their workplace.

### Lessons Learned

Development of new physician or patient facing products that are designed to facilitate communication and care need to include a number of factors and follow a few basic design principles. As suggested by Kreuter and Dearing and Brownson and colleagues [16,17], using the Designing for Dissemination and Implementation (D4D&I) principles can increase the likelihood that the final product will be adopted, implemented and used in a sustained manner. Based on one of the D4D&I principles, a key lesson learned from the small-scale implementation study was engaging various stakeholders (ie, patients, physicians, caregivers, health care system leaders) early in the project (ideally in the development of the study design) and continually engaging with these groups throughout the study. Gaining support and input from those who will not only use the tool (the end user), but also those who will support the end user is essential to ensure utilization and satisfaction. Similarly, while engagement is a continual process, so is the iterative process in the development of the end product. The end product should evolve based on the needs of and testing by the end users. Patients experiencing a cancer diagnosis can have a vast health care team, including specialists, pharmacists, navigators, and nurses. As a result, the development, testing, and implementation of a decision aid needs to have input from an interdisciplinary team as well as the patients they serve.

Another important factor in designing for dissemination and implementation is to understand what barriers and facilitators exist for the implementation of these aids in the health care setting and what additional resources are needed to make their implementation successful. In our study, we collected information on barriers and facilitators of local adoption and implementation (eg, exiting channels, processes, and provider preferences). However, more work needs to be done to further explore the multilevel context in which these decision aids are implemented and used in a sustainable manner. Tools like the one developed as part of the My Own Health Report study for the pragmatic, mixed methods, and multilevel assessment of context can support such data collection [18].

### Limitations

This study is not without limitations. The small-scale implementation trial was conducted in a small number of settings with little geographic diversity. Expanding to a large number of clinics across more diverse settings and patient populations may have provided different utilization patterns and better integration into practice. All of these settings had electronic health records, and thus providers would have liked the tool integrated into the system for ease of use. Testing the use of this tool in clinical settings that do not have an electronic health record or resource poor environments in terms of decision aids and decision support tools might have provided different results. Further exploration is warranted.

### Future Implications

As a result of the efforts described in this paper, 2 major steps were taken. Given the major impediments to deploying a tool like this in a clinical setting, and the strong movement towards open access to health information, a decision was made to turn SEER*CSC into a public-facing application. Given the considerable use of technical medical language necessary to describe a tumor, this has required extensive revisions to the user interface to explain terms and make the overall language more understandable to a general audience. In addition, appropriate language on intended use and disclaimers must be added. Work on this is underway. Second, while no single tool can address all questions and with more tools being made available, it can be quite confusing to both physicians and patients which tools are most appropriate for which situations. The National Cancer Institute is supporting pilot work to integrate sets of high quality tools so their appropriate use case in the clinical decision pathway is clearer (eg, just after diagnosis, after initial surgery and prior to adjuvant therapy, and after a relapse).

Web-based prognostic tools face major challenges as they compete with many other priorities for the time of health care professionals. Streamlining their use (eg, by incorporation into EHRs), making sure there is institutional support, and making available information that is immediately actionable may all be necessary but not always sufficient conditions for their widespread acceptance. Making tools available to the general public faces challenges such as overcoming technical language necessary to describe the extent of disease, making sure that the tool and its limitations are properly understood, and avoiding discouraging patients with poor prognosis from having hope. The development of meta-tools for understanding the strengths and limitations of various tools and the place of each in the clinical decision making pathway are necessary. Despite these major obstacles, prognostic tools are important instruments to make sure evidence-based medicine makes its way into clinical practice and the shared decision-making conversation.

## Abbreviations

SEER*CSC: Surveillance, Epidemiology, and End Results Cancer Survival Calculator
EHR: electronic health records
NCI: National Cancer Institute
